# Industrial Design education in Australia: a competence analysis across primary, secondary and tertiary education levels

**DOI:** 10.1007/s10798-023-09822-0

**Published:** 2023-03-21

**Authors:** Kathryn Deighton, Blair Kuys, Shivani Tyagi

**Affiliations:** 1 Centre for Design Innovation, Swinburne University of Technology, Melbourne, Australia; 2 School of Design and Architecture, Swinburne University of Technology, John Street, Hawthorn, VIC 3122 Australia

**Keywords:** Industrial Design, Design, Education, Competences, 21st century, Value

## Abstract

Industrial Design education is poorly understood by laypeople but is present in Australian curricula from primary through to tertiary education levels. Designers and design researchers have long recognised the value of the broad-ranging skills, knowledge fields, and personal qualities design education imparts, but this understanding is generally not shared by the wider community who may see design as surface decoration. This research identifies indicators of value and relevance taken from the twenty-first century competences literature, then measures their presence in four different Industrial Design education settings. Two studies were undertaken. First, Industrial Design educators from primary, secondary, and tertiary levels were surveyed. Then diverse Industrial Design education stakeholders from education and non-education settings were interviewed. The studies gathered both quantitative and qualitative data on the value and relevance of current Industrial Design education in Australia. The result is a comprehensive analysis of the twenty-first century competences present in Australian Industrial Design education, which concludes with recommendations for ways Industrial Design education can benefit twenty-first century learners, as well as ways it should evolve to remain relevant.

## Introduction

Industrial Design education is poorly understood by policy makers and the general public (Australian Government, 2018; Driver et al., 2011; Goatman & Moodie, 2014) but exists in Australian curricula from primary through to tertiary education levels.

Designers and design researchers see Industrial Design as an intellectual undertaking that is complex, future focussed (Jonas, 1999; WDO, 2018); and inseparable from ethical (Nelson & Stolterman, 2012), environmental (Papanek, 1985), and cultural contexts. They see that it has a broad knowledge base (Buchanan, 1990; Papanek, 1985), and a powerful capacity for creative problem solving (Archer, 1964/1984; Buchanan, 1990; WDO, 2018). Whereas members of the public and policy makers may see it as surface decoration (Driver et al., 2011; Norman, 2017). This research seeks to understand the true value of Industrial Design education.

Wright et al. (2013) identify a paucity of research measuring the value of design and communicating this value to policy makers. While the extensive twenty-first century competences literature describes skills, knowledge, and personal qualities that will be most valuable in upcoming decades (Anderson, et al., 2017; Hajkowicz et al., 2016; ISA, 2017; Leadbeater, 2016; OECD, 2019; Voogt & Roblin, 2012; WEF, 2018). This research used the skills, knowledge, and personal qualities from the established twenty-first century competences literature to measure the value and relevance of current Industrial Design education in Australia. The aim was to develop a resource for policy makers and researchers which could influence important decisions about Industrial Design education and public projects over coming decades. This research aimed to enable decision makers in the community to better understand what Industrial Design education has to offer. It also aimed to enable current Industrial Design stakeholders to understand ways Industrial Design education could be improved.

The research measured the presence of key twenty-first century competences in current Australian Industrial Design education settings using both a rating survey and semi-structured interviews. The rating survey was completed by 46 Industrial Design educators from primary, secondary, vocational, and undergraduate university education levels. Then semi-structured interviews were conducted with 23 Industrial Design education stakeholders. Four specific education levels were studied for two reasons. Firstly, Industrial Design education has not been examined across such broad education levels before; even though foundational impacts on later education outcomes are well known in other fields (DET, 2016; Education Council, 2019). Secondly, the developmental and structural contrasts between these four education levels would likely foster different types of value and relevance. Therefore, research results were likely to suggest structural adjustments that could improve Industrial Design education across many levels.

The scope of the research was limited to Australian data sources. However, the findings are relevant to other regions, particularly those with a similar design culture and economic climate to Australia. Nevertheless, we acknowledge that relevance to some regions may be unknown and unknowable (Sen & Sharma, 2011).

This research finds that Industrial Design education is likely to impart strong capabilities in a range of key twenty-first century competences. It finds that Industrial Design education may be improved through more overt teaching of *collaboration* skills, the nurturing of further *entrepreneurial skills,* and further inclusion of emerging technologies such as virtual reality (VR), augmented reality (AR), artificial intelligence (AI), the internet of things (IoT), and generative design software. This research finds that Industrial Design education can be used to deliver vital twenty-first century competences from primary school level through to university level, and that its value be recognised through grants and public funding.

## Literature review

This literature review first defines the terms *competence* and *personal qualities*, as used in this research. The purpose of Industrial Design education is then examined using selected historical and current sources. Next, significant projections about mid-late twenty-first century life are summarised and used to predict activities that Industrial Designers may be involved in over coming decades. Finally, the most in-demand twenty-first century competences and the top Industrial Design competences are identified and compared.

### The terms “Competence” and “Personal Qualities”

The established twenty-first century competences literature is used in this research to benchmark the value and the twenty-first century relevance of current Industrial Design education in Australia. Voogt and Roblin (2012), use the term *twenty-first century competences* to refer to twenty-first century-relevant skills, knowledge, and attitudes applicable to many fields, on the basis that it is used in many academic papers and government reports (Voogt & Roblin, 2012). The term has also been adopted by the European Parliament (European Union, 2018). The term *twenty-first century competences* is therefore adopted for this research, except that in this research it refers to skills, knowledge, and *personal qualities*. The term *personal qualities* is used in this research to refer to “non-cognitive” characteristics such as, commitment, motivation, confidence, curiosity, perseverance, self-awareness, resilience, self-esteem, adaptability, and empathy, in line with Duckworth and Yeager (2015).

### The purpose of industrial design education

As with all fields of education, Industrial Design education should support the development and wellbeing of the whole person for work and for life (Education Council, 2019; OECD, 2018), for the benefit of individuals (*Basic Education Act* (Fin); Education Council, 2019; Plutarch, 1st Century AD-a; United Nations, 1948), and for the enrichment of their local and global communities (Education Council, 2019; World Bank, 2018). Industrial Design education should produce capable, passionate, lifelong learners (Durrant-Whyte, 2015; Education Council, 2019; Hajkowicz et al., 2016; World Bank, 2018) who can think and act for themselves (Duckworth, 1964; Plutarch, 1st Century AD-b). Industrial Design education has expanded from the art, science, and technology of the Bauhaus (Bauhaus, 2021; Findeli, 2001) into a discipline that must also manage “complex and innovative processes involving science, technology, society, business models, marketing, and political issues” (Collina et al., 2017, p. S1002).

### Impacts of upcoming global conditions on the industrial design profession

Global conditions in the next decades will influence which Industrial Design competences are the most valuable and relevant. Projected conditions include the likelihood of extreme technological development (Frey & Osborne, 2017; Hajkowicz et al., 2016; Kurzweil, 2001; WEF, 2017), pressing environmental sustainability concerns (EEA, 2015; Meadows et al., 1972; Turner, 2008; WWF, 2016), and evolving, complex interactions between social, political, environmental, economic, and technological factors (Davidson, 2005; Daly, 2012; Meadows et al., 1972; Steward Redqueen, 2016; Turner, 2008; WWF, 2016).

As a result of global conditions of upcoming decades, future Industrial Designers will need to be lifelong learners (Hajkowicz et al., 2016; Scott, 2015; WEF, 2016a; World Bank, 2018) who must keep abreast of changing conditions, especially in relation to emerging technologies, including green technologies. Future Industrial Designers will need to be mindful of resource and energy use when designing for the rising global middle classes (WEF, 2016a; WEF 2018). They will need to do more with less, and even try to design commercial goods that have an overall regenerative effect on the biosphere (Ellen Macarthur Foundation, 2021). It is likely future Industrial Designers will help develop innovative ways to feed the world’s growing population (EUFIC, 2018) through involvement in the design of 3D printed foodstuffs, more efficient packaging, influence over food trends, and more efficient production and transport systems (EUFIC, 2018; Hunter et al., 2017). Furthermore, they will need to be systems thinkers (Willis, 2012), and apply designerly ways of thinking (Cross, 1982) to the management of rising complexity and uncertainty (Rittel & Webber, 1973/1984; Schön, 1983; Simon, 1973/1984).

Appraisal of twenty-first century employment forecasts suggests that Industrial Designers of coming decades will need to work in more flexible ways and will need to compete globally in their work (Hajkowicz et al., 2016). Industrial Designers of coming decades will have to consider the needs of ageing users (UN, 2019), and will work with ageing colleagues (Hajkowicz et al., 2016). They will need to be educated about Industry 4.0, and emerging technologies such as the Internet of Things, machine learning, digital trade, augmented and virtual reality, advanced manufacturing, new materials, wearable electronics, 3D printing, autonomous transport, robotics, and biotechnology (CEDA, 2015; WEF, 2018). This knowledge will need to be applied to the design of both physical and virtual products. Industrial Designers of coming decades will also need to have a sound understanding of the privacy, security, intellectual property, and health and safety implications of all design outcomes (Atzori et al., 2014). These knowledge requirements suggest that Industrial Designers of coming decades will need increased levels of scientific understanding. They will also need to be resourceful in the face of geopolitical disturbances such as the COVID-19 pandemic, because of the low resilience of design professions to adverse conditions (ABS, 2018; NSC, 2020). However, more manufacturing may move back to Australia post-pandemic (Australian Government, 2020) and this may have a positive effect on Industrial Design work prospects. Industrial Designers of coming decades are also likely to need to reskill often as their jobs evolve (WEF, 2020) or they may need to move their skillsets to related or even unrelated disciplines (WEF, 2020).

To better understand what is required, we provide a comparison between the top competences from the twenty-first century competences literature mapped alongside the top competences from the Industrial Design competences literature as summarised in Table 1. Table 1 was generated through analysis of the literature on twenty-first century competences and the literature on competences required in Industrial Design learning. These bodies of literature were identified by selecting relevant articles obtained from the search terms of “twenty-first century education”, “twenty-first century competences”, “twenty-first century skills”, “industrial design competences”, “industrial design education”, and similar. Grey literature, including government reports and reports from international organisations on these same topics was also reviewed.

**Table 1:**
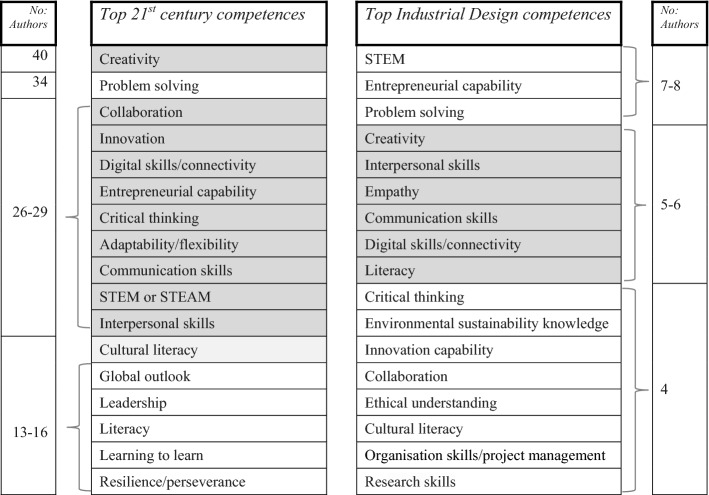
Top twenty-first century competences and top industrial design competences

The Top twenty-first century competences listed here were sourced from 55 texts. The Industrial Design competences listed here were sourced from 13 texts

The references used to generate Table 1 can be viewed in Appendix 1 and Appendix 2. Competences at the top of the table were endorsed by a large proportion of authors, and competences close to the bottom of the tables were endorsed by a smaller yet still substantial proportion of authors.

Comparison of the twenty-first century competences and Industrial Design competences reveals that many of the same competences are valued by twenty-first century competences authors and Industrial Design competences authors. There are four exceptions to this generalisation. These are that *empathy*, *technological knowledge*, *environmental sustainability knowledge*, *ethical understanding*, *organisation skills/project management,* and *research skills*, are endorsed more by the Industrial Design competences authors. This suggests a higher than required tendency towards technology, efficiency, environmentalism, investigation, and social conscience in Industrial Design learning. *Adaptability & flexibility* is endorsed less by the Industrial Design competences authors despite its prevalence in conventional design approaches. *Leadership* is endorsed far more by the twenty-first century competences authors than the Industrial Design competences authors, suggesting that Industrial Designers are not necessarily expected to be leaders. Finally, surprisingly, *global outlook* is mentioned by a higher percentage of twenty-first century authors than Industrial Design competences authors, despite much reliance on offshore manufacturing by many Industrial Designers globally. Two final observations from the literature are firstly, that the top Industrial Design competences are moderately more likely to be hard skills or hard knowledge fields. Hard skills are codifiable and measurable skills that can be taught explicitly (Oxford Reference, 2021) such as language learning, arithmetic and machine operation. This suggests that technical, scientific, and practical competences are important aspects of Industrial Design.

Secondly, the Industrial Design competences authors place a somewhat higher emphasis on *entrepreneurial capability* than the twenty-first century competences authors. This may result from Industrial Design being so closely affiliated with the manufacturing of saleable goods. This comparative analysis of the twenty-first century competences literature and the Industrial Design competences literature will be compared with the final results of this research.

In summary, the literature indicated that the top twenty-first century competences are a broad and variable range of skills, knowledge, and personal qualities, and that there is a substantial crossover between these competences and those valued within the Industrial Design competences literature. Industrial Designers of coming decades will need to adapt to extreme technological changes, pressing environmental concerns, and complex, evolving systems involving social, political, environmental, economic, and technological factors. Industrial Designers of coming decades are likely to need to tackle problems related to food security, the ageing population, increasing global populations, privacy, security, health, and safety. There are likely to be both positive and negative impacts on Industrial Designers as a result of the COVID-19 pandemic and other potential geopolitical disturbances. Gaps identified in the literature were that: no studies followed the progression of Industrial Design education from school level through to tertiary level; and no studies had quantified the value of Industrial Design education in holistic ways. This research therefore aimed to analyse different levels of Industrial Design education and to quantify the value of Industrial Design education in ways that did not rely on economic measures. The research questions addressed in this research were, *What is the value and twenty-first century relevance of current Industrial Design education practice across primary, secondary, vocational, and undergraduate university education levels in Australia?* and, *How can these Industrial Design education levels improve their twenty-first century value and relevance?*

## Method

Two research methods were used to investigate the research questions. Firstly, a cross-sectional rating survey was completed by educators. The rating survey was designed to quantitatively measure the value and twenty-first century relevance of students’ Industrial Design projects. Secondly, semi-structured interviews were conducted with a broad range of Industrial Design stakeholders. The interviews were designed to collect data that could be easily compared with the survey data and to provide qualitative insights into the value and relevance of Industrial Design education in Australia. These methods are further described shortly.

### Compliance with ethical standards

The survey and interviews were conducted in line with the *National Statement on Ethical Conduct in Human Research,* and were approved in August, 2019 (SHR Project 20,211,215-6629). A separate ethics application was also approved by the Victorian Government Department of Education and Training (DET Project 2019_004141). We acknowledge the support provided by the Victorian Government Department of Education and Training. All participants’ data in the survey and interviews were anonymised or were anonymous from the beginning, since no signifiers were used. The participants agreed to undertake the study based on the informed consent statement at the commencement of the survey and interview. As such, they gave consent for the data to be used for this research.

### Participants across both studies

Participants in the two studies were Australian Industrial Design education stakeholders and one Indonesian international student studying Industrial Design in Australia.

### Survey study participants

Forty-six survey responses were gathered from 19 university lecturers, six vocational education educators, 15 secondary school teachers, and six primary school teachers. They were all teaching project-based Industrial Design projects (or 3D design projects at primary level) in their classes. Focusing on educators in the survey study allowed a comparison of the actual class contents across these four education levels.

Specific education levels were targeted in order to understand variations arising from developmental differences, from structural aspects of the education settings, and from the differing purposes of the education levels. At primary school level, grade 5 and/or 6 was selected as a group that could understand the purpose of design and design processes in some depth, may lack abstract thinking skills (Piaget, 2016), and would have comparatively low assessment pressures. At secondary school level, year 11 and/or 12 was selected as a group that was able to specialise in design, possessed abstract thinking skills (Piaget, 2016) and adult-level psychomotor skills (Cratty & Noble, 2016), and was affected by high-pressure assessment systems. At the vocational education level, 1st or 2nd year level was targeted as a group where a high degree of specialisation applied, workplace and hands-on skills were emphasised (Norton et al., 2018), strong government regulation affected assessment structures (Parliament of Australia, 2018), and students were of an age likely to be interested in questions of responsibility and challenging the status quo (Kohlberg, 2010; Loevinger, 1973). While at university level, 3rd and/or 4th year was targeted as a group where a very high degree of specialisation was possible, and where students were likely to have comparatively higher theoretical and academic aptitudes (Norton et al., 2018). This university level was also seen as a level where students would be affected by the autonomy brought about by self-regulation of university courses (Norton et al., 2018), and where students would have an even higher interest in questions of responsibility and challenging the status quo than the vocational education students due to their slightly higher age range (Kohlberg, 2010; Loevinger, 1973).

### Survey materials

The research instrument used for the survey was a rubric-style rating tool hosted on the *Qualtrics* online survey platform. The survey was structured as a series of five-point rating scales measuring the presence of the top twenty-first century competences. Basic demographic information was collected, and two text entry questions asked for the “project title [of the student project educators chose to respond to the survey about]”, and “any further comments”.

In the survey, ten of the thirteen top twenty-first century competences from the literature were rated by educators according to their level of presence in their students’ class projects. These ten twenty-first century competences were: creativity, problem solving, collaboration, innovation, digital skills/connectivity, entrepreneurial capability, critical thinking, adaptability/flexibility, STEM or STEAM (Science, Technology, Engineering, [Art], Mathematics), and cultural literacy. The top twenty-first century competences of communication skills and interpersonal skills were excluded from the survey because retrospective rating of these two competences would have been too unreliable. Finally, the competence of environmental sustainability knowledge was added to the rating list because it had been so prevalent in the literature on twenty-first century life (EEA, 2015; Meadows et al., 1972; Turner, 2008; WWF, 2016). Consequently, the final list of 11 competences rated in the survey were:

- *creativity*
- *problem solving*
- *collaboration*
- *innovation*
- *digital skills/connectivity*
- *entrepreneurial capability*
- *critical thinking*
- *adaptability/flexibility*
- *STEM or STEAM (Science, Technology, Engineering, [Art], Mathematics)*
- *cultural literacy*
- *environmental sustainability knowledge*

Competences were sometimes rated individually but were often split into between two and five sub-competences that were easier for respondents to identify. For example, the competence of *collaboration* was divided into the sub-competences of *idea &/or resource sharing in a student group or student partnership*, *role/responsibility taking in a student group or student partnership,* and *co-design &/or co-decision making amongst students*. Table 2. shows an example rating question from the survey. It shows the question used to rate one of the three *collaboration* sub-competences, *idea &/or resource sharing in a student group or student partnership*.

**Table 2:** Example rating question from the survey

Q1a Collaboration 1 Idea &/or resource sharing in a student group or student partnership				
1. Not applicable or not present: The top few projects did not include idea sharing or resource sharing within student groups or student partnerships	2. Slightly present: The top few projects used a small amount of idea sharing &/or resource sharing in student groups or student partnerships, e.g. minor team brainstorming, a small amount of peer feedback, a very small amount of sharing of research materials	3. Moderately present: The top few projects used a reasonable amount of idea sharing &/or resource sharing in student groups or student partnerships, e.g. some team brainstorming, meaningful peer feedback, team researching, &/or shared materials	4. Strongly present: The top few projects used a significant amount of idea sharing &/or resource sharing in student groups or student partnerships, e.g. team members built on each-others' ideas in several ways, research and materials were shared, &/or there was meaningful and useful team brainstorming	5. Extensively present: The top few projects used idea sharing &/or resource sharing extensively in student groups or student partnerships, e.g. team members built on each-others' ideas in many ways, research and materials were freely and frequently shared, &/or there was energetic &/or fruitful team brainstorming

### Survey procedure

The survey procedure was as follows. The authors used publicly available contact information to get in touch with school principals and tertiary education leaders, then asked them to forward survey links to relevant educators in their institutions. After surveys were completed, data were downloaded from the *Qualtrics* platform to a password protected laptop. The data were next graphically represented and then analysed by comparing the patterns of high ratings, moderate ratings, and low ratings across the different competences and across the different education levels. The surveys were completed between November 2019 and March 2020 before the first pandemic lockdowns affected education delivery in Australia. Survey results will be reported shortly.

### Interview study participants

Twenty-three Industrial Design education stakeholders were interviewed. Because the survey was limited to educator viewpoints, the interviews were designed to complement the survey data by capturing the viewpoints of a broad range of Industrial Design education stakeholders. The stakeholders included educators, students, education leaders, curriculum and assessment professionals, design teacher association representatives, professional Industrial Designers, and a makerspace librarian. Where interviewees were associated with education institutions, the education levels they came from were identical to those used in the survey study, namely grade 5 and/or 6, year 11 and/or 12, 1st or 2nd year vocational education, or 3rd or 4th year undergraduate university. This was done to facilitate comparison between the two studies.

### Sampling strategy

A combination of cluster sampling and judgemental sampling was used to recruit interviewees. Judgemental sampling was used to identify a diverse set of Industrial Design education stakeholders likely to provide rich information or interesting viewpoints. The non-random technique of judgemental sampling was justified by the fact that the intention of the interview study was to collect a snapshot of diverse views from a particular set of Industrial Design education stakeholders at a particular point in time. Clusters of participants were recruited from schools and tertiary institutions, both for the sake of efficiency, and as a way of comparing different viewpoints about the same system.

### Interview questions

A set of two leading questions was used in the semi-structured interviews. These were designed to elicit authentic opinions that could answer the research questions. The interview questions were,

What knowledge, skills and personal qualities do you think Industrial Design education gives or should give to a student?,

and

What do you think Industrial Designers and Industrial Design education can contribute to a community or society both now and in the future?

Both questions were designed to discover respondents’ authentic thoughts about what competences are inherent in Industrial Design learning, as well as what Industrial Design-related competences are most relevant for the twenty-first century. The interviewer only asked further questions to draw out details and explanations from interviewees.

### Interview procedure

All interviewees consented to have their interviews recorded except for one, whose interview was documented through notetaking and whose final interview document was emailed to them for verification. Notes were also taken during all other interviews. Consent documents for the interviews were used to collect a small amount of demographic data about the interviewees. Interview recordings were transcribed and analysed quantitatively and qualitatively with the help of *NVivo* software.

### Analysis

Mixed methods analysis was used. Both the rating survey and the semi-structured interviews were analysed quantitatively so that they could produce clear and comparable data sets. The semi-structured interviews were also analysed qualitatively in order to deepen understanding of the reasons behind the quantitative results and to uncover emergent new knowledge. Two text entry questions from the survey were also analysed qualitatively.

### Survey analysis

The survey results were analysed as follows. Five-point scale rating data from the online survey platform were exported into spreadsheet software. The data were simplified into high ratings (*extensively present or strongly present* ratings), moderate ratings (*moderately present* ratings), and low ratings (*slightly present, not present or not applicable* ratings) across the different competences and across the different education levels. They were then arranged into a series of 100% stacked bar charts. One hundred percent stacked bar charts were chosen as the main analysis and communication tools for this research because they had been successfully used to communicate to a broad audience in a comparable report (WEF, 2018).

A formative model was used to interpret the survey data. For example, where general competences such as *creativity*, were divided into several sub-competences, such as, *fluency of idea generation*, *uncommon & original ideas*, and *flexibility in idea generation*, a contribution from each sub-competence was taken to establish an overall value for the main competence.

Demographic data were tabulated and interpreted. Furthermore, the “project title” and “any further comments” data from the rating survey were analysed qualitatively by thematic analysis in order to identify unexpected insights and learn more about likely levels of error in the data.

### Survey results

**Fig. 1:**
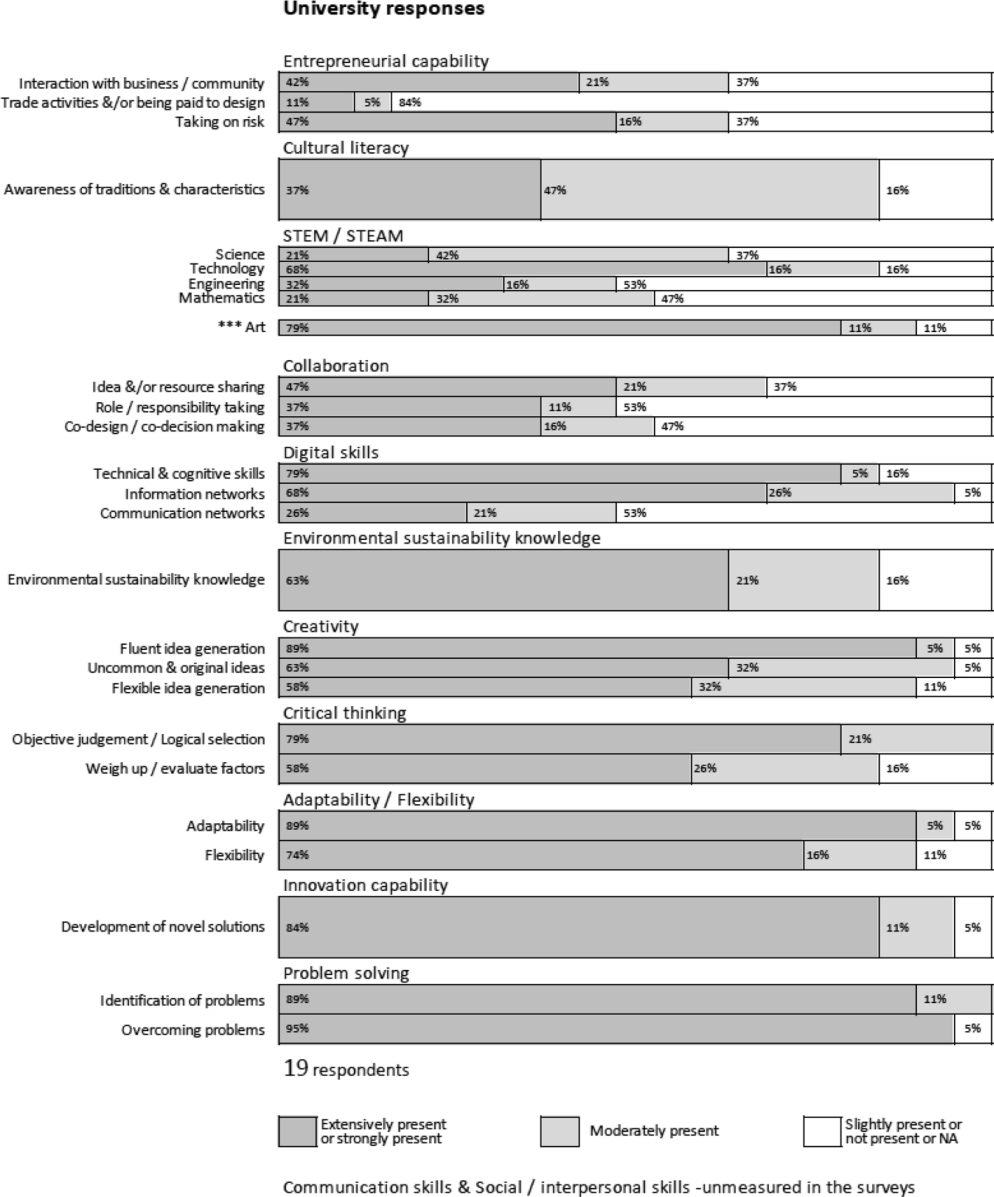
Overview of survey results from university cohort, showing high, low, and moderate responses for each sub-competence

**Fig. 2:**
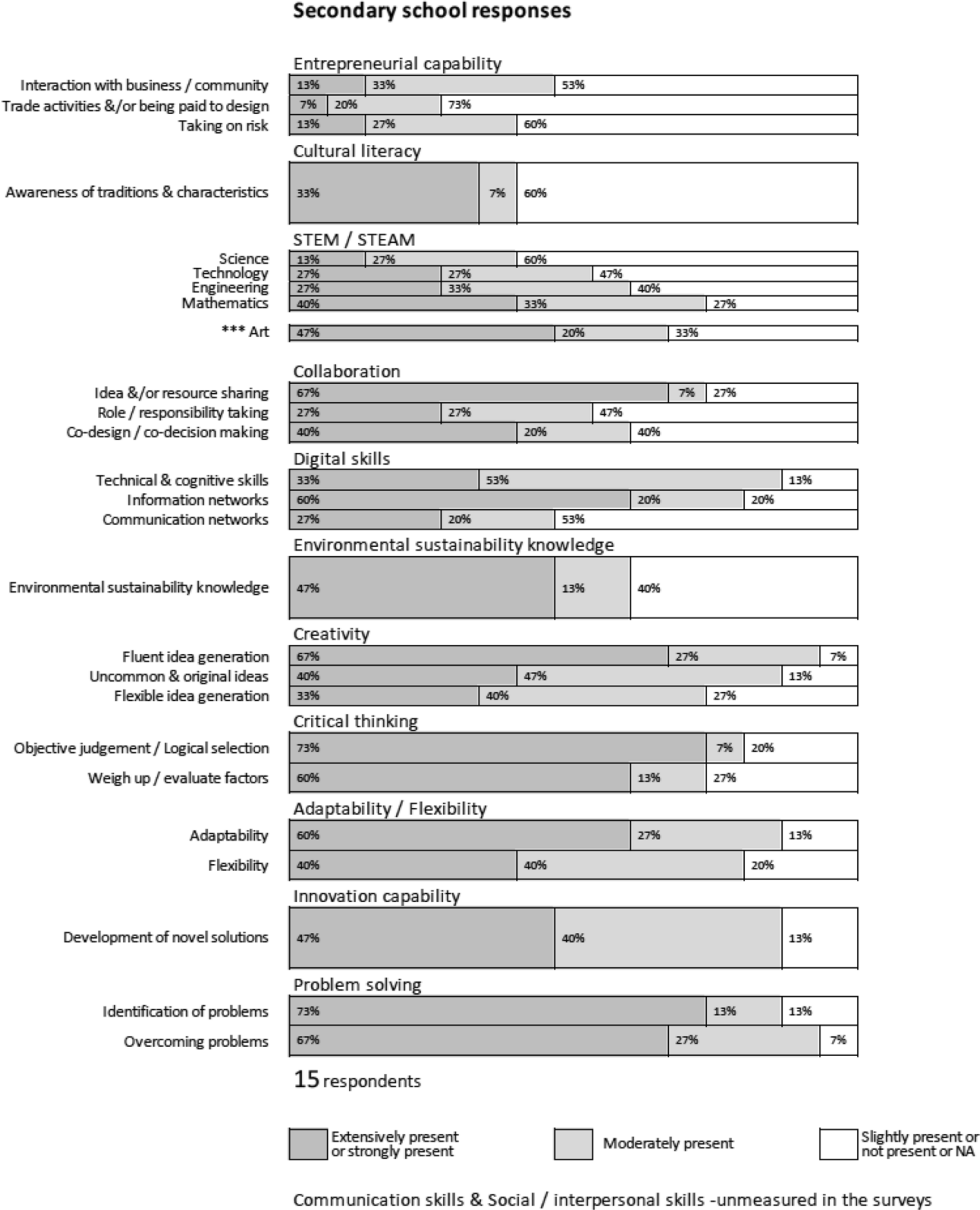
Overview of survey results from secondary cohorts, showing high, low, and moderate responses for each sub-competence

The university lecturer survey cohort was the only survey cohort to produce somewhat representative and generalisable data without unexpected demographic biases. There were 19 university lecturer respondents. This was estimated to be around 50% of that target population. The remainder of the survey study was described as exploratory. The 15 secondary teacher responses were given some weight in analysis because an *SPSS* mock MANOVA analysis had indicated that this number was only one short of being statistically meaningful. Values entered into the mock MANOVA analysis were for 11 outcome measures and four groups. G-Power was used assuming a significance of 5%, power of 80% and a large effect size (eta-squared = 0.14). However, it was noted that the secondary cohort was biased towards certain respondent types as seen in Table 3. The vocational education and primary teacher cohorts could only be analysed superficially because these cohorts returned just six responses each. Therefore, the vocational education and primary teacher survey data will not be discussed in this paper. Table 3. summarises the university and secondary survey respondent demographics and Fig. 1 and Fig. 2 show the 100% stacked bar charts that were used to analyse and communicate the university and secondary educator survey data.

**Table 3:** Demographic data collected in the survey

	Australian state	City or regional	Subjects reported on	Female, Male or X
University lecturers	5 States	19 C 0 R	PBL in a tertiary Industrial Design	5 F
	1 ACT		or Product Design course	13 M
	8 NSW		19 (All)	1 None
	3 Qld			
	2 SA			
N = 19	5 Vic			
Secondary school teachers	2 States	9 C 6 R	Design technology subject 10	4 F
N = 15	3 NSW		Design subject 4	11 M
	12 Vic		IB Design technology (Science emphasis) 1	

SA = South Australia, WA = Western Australia, PBL = Project Based Learning, IB = International Baccalaureate

Overall, the survey results suggested substantial value and twenty-first century relevance in university level Industrial Design learning in Australia. They suggested relatively high value and twenty-first century relevance in the secondary-level Industrial Design settings studied. The university respondents had the strongest alignment with the twenty-first century competences in all cases except in the *collaboration* competence, where alignment was similar in the university and secondary cohorts. In the university survey cohort, the competences demonstrating particularly strong alignment with the top twenty-first century competences were *problem solving, innovation capability, adaptability/flexibility, critical thinking, creativity*, and *environmental sustainability knowledge*. Strong alignment was defined as a competence where 60% or more of respondents reported strong or extensive use, and/or fewer than 20% reported low or non-existent use. Sub-competences that showed strong alignment with the top twenty-first century competences in the university lecturer cohort were the *digital skills* sub-competences, *technical and cognitive [digital] skills*, and *information networks*, and the *Science Technology Engineering Arts Mathematics (STEAM)* sub-competences of *Technology skills and knowledge*, and *Art skills & knowledge*.

The sub-competences demonstrating low alignment with twenty-first century competences in the university cohort, defined as sub-competences where more than 50% of the university lecturer respondents reported low or non-existent use, and/or fewer than 33% reported high or extensive use, were *[digital] communication networks*; the *Science Technology Engineering Mathematics (STEM)* sub-competences of *Science skills & knowledge*, *Engineering skills & knowledge*, *Mathematical skills & knowledge*; and the *entrepreneurial capability* sub-competence, *trade activities and/or being paid to design*. Additionally, *collaboration* and *Mathematics skills* were evident at varied levels across the cohort, and *cultural literacy* seemed to be important only at a basic level.

In the secondary teacher data set, competences demonstrating strong alignment with twenty-first century competences, according to the definitions used above, were *problem solving,* and *critical thinking.* Sub-competences showing strong alignment with the top twenty-first century competences were *adaptability*; the *creativity* sub-competences, *fluent idea generation,* and *uncommon & original ideas*; the *collaboration* sub-competence of *idea &/or resource sharing*; and the *digital skills* sub-competences, *technical & cognitive [digital] skills*, and use of *information networks*. The competences showing low alignment with the top twenty-first century competences in the secondary cohort were *cultural literacy* and *entrepreneurial capability.* Sub-competences with similarly low alignment in the secondary teacher cohort were the *collaboration* sub-competence of *role/responsibility taking in a group or partnership*; the *digital skills* sub-competence of *communication networks*; and the *STEM* sub-competences, *Science skills & knowledge*, *Technology skills & knowledge*, and *Engineering skills & knowledge*. Additionally, *cultural literacy*, *environmental sustainability knowledge*, *collaboration*, *technology skills*, *Engineering skills*, and *entrepreneurial capability* were found at very variable levels across the cohort, suggesting they were used very differently by different secondary teachers.

### Text entry questions

The “project title” question asked educators to specify the title of the student project they were responding to the survey in relation to. Analysis of responses revealed that functional and technological projects with specified project outcomes were more prevalent in the secondary school cohorts, and open-ended and socially themed projects were more prevalent in the university cohorts. This may have resulted from different levels of psychosocial, psychomotor, and moral development between levels. Higher education levels would be expected to be drawn towards social and political projects (Kohlberg, 2016), and younger education levels may have needed to develop technical skills before applying them in creative ways (Cratty & Noble, 2016).

### Differences between the cohorts

The reason the university cohort aligned more strongly with the top twenty-first century competences than the secondary cohort was not clear, as the survey had been designed to apply equally to all education levels studied. One possible explanation is that more complex projects are able to be run with older students than with younger students (Girgis et al., 2018). However, at senior secondary level, many Australian Design curricula run for a whole semester (Board of Studies NSW, 2013; QCAA, 2018; VCAA, 2017a; VCAA, 2017b) and senior secondary students should be capable of using many competences in that time. Further explanations for this finding were sought in the interview study.

### Effects of biases and sources of error

There were a number of known biases affecting the rating data. The most obvious one arose from differences between the priorities of different secondary curricula and the different backgrounds of people teaching the secondary subjects. For example, secondary education results were skewed towards the “technical” and “making” priorities of Design Technology teachers and away from the more artistic priorities of Design teachers. Related but unknown biases would have arisen from the tertiary Industrial Design curricula represented in the study. This is because university respondents would have been following diverse curricula as a result of the self-regulation of Australian universities (Norton et al., 2018) and as a result of the inclusion of lecturers’ specialist knowledge in their own curricula.

Additionally, all the survey responses were biased towards the views of male respondents, city-based respondents, respondents based in NSW or Victoria, and Industrial Design and Design trained respondents. (Although many of these biases are likely to be representative of these cohorts.) Errors also resulted from inaccurate reading of survey instructions, variations in rating leniency amongst respondents, question order effects, demand biases, and social desirability biases.

It was anticipated that the interview study would provide triangulating data to validate the survey results. It was also anticipated that the interview data would provide insights into the relationship between what was physically produced in classes (as expressed in the surveys), and what was theoretically valued by stakeholders (as expressed in the interviews).

### Interview analysis

Analysis of the interview data was primarily conducted using quantitative content analysis. This was done to ensure comparability between the survey study and the interview study. Some qualitative thematic analysis was also undertaken. Analysis was grounded in comparison with the existing twenty-first century competences literature, the existing Industrial Design competences literature, and the survey study. A hierarchical coding structure was developed in *NVivo.* This involved the creation of up to four hierarchical levels of grouped codes. Analysis of the final coding structure also involved cross referencing with the demographic data.

### Interview results

The duration of interviews ranged from 11 to 66 min, with the majority of interviews going for between 18 and 44 min. As can be seen in Table 4, opinions expressed in the interviews were more likely to come from females in the young adult or 45–54 age ranges. They were moderately more likely to relate to the secondary schooling context, and were much more likely come from a Victorian, city-based location. These biases resulted by chance, as interviewee recruitment was based partly upon who held the job roles targeted for the study, who agreed to be interviewed, who was able to be contacted, and which students were put forward by institutions as possible interviewees. Table 4 shows the demographic attributes of the interviewees.

**Table 4:**
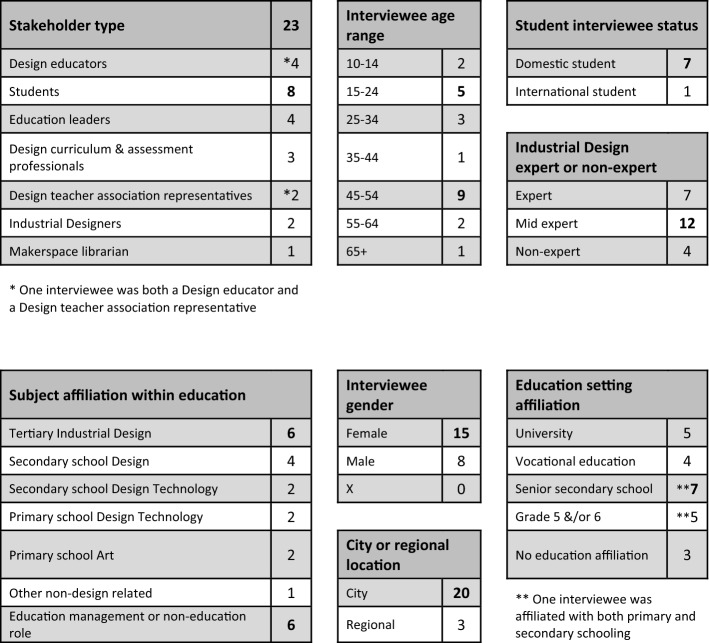
Interviewee demographics

**Fig. 3:**
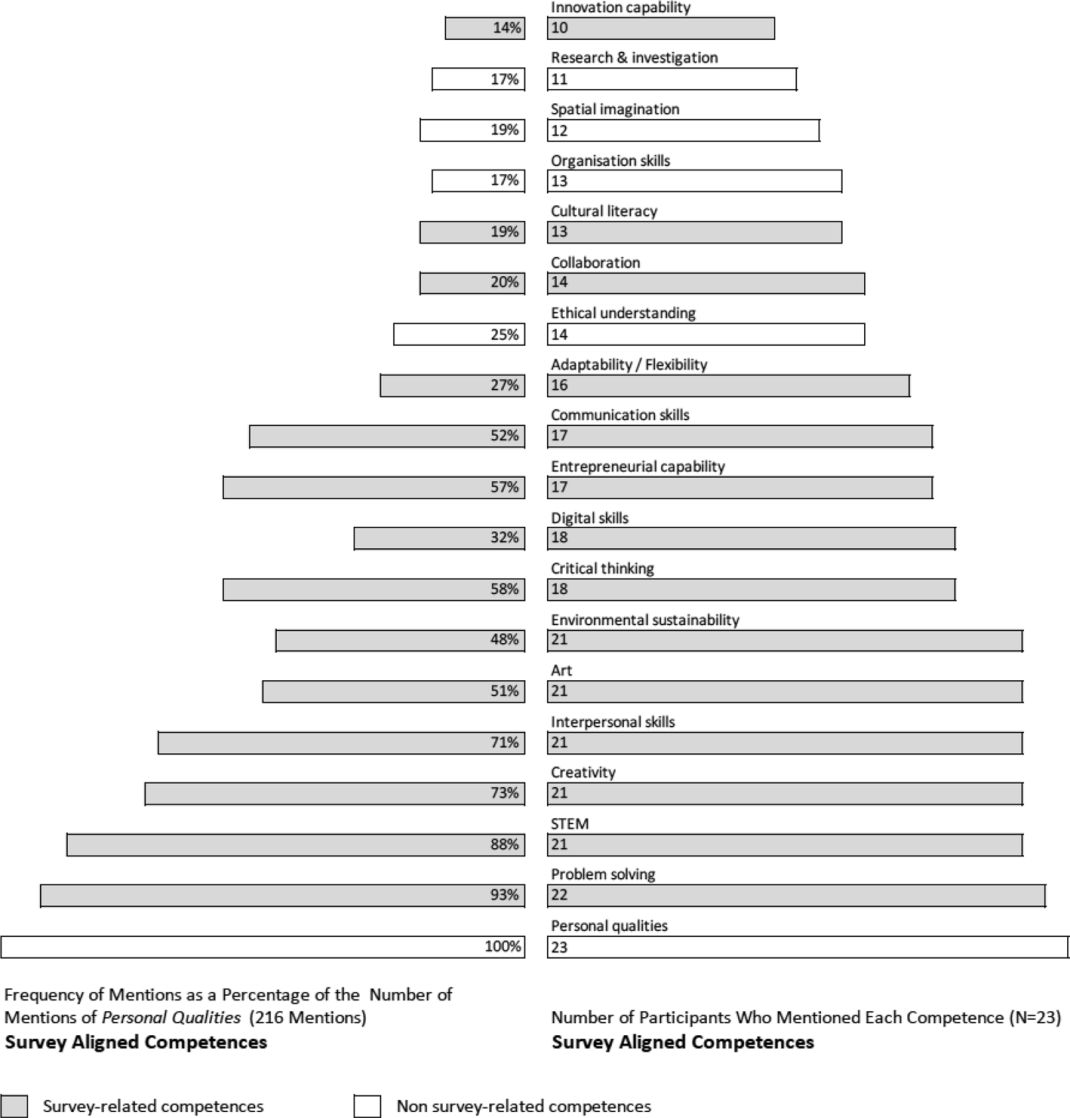
Summary of key interview data

The interview content analysis measured the number of interviewees mentioning each survey-related competence in combination with the number of times they mentioned them. It also identified any competences that were discussed as frequently as the survey competences. Figure 3 summarises the 14 most discussed survey-related competences in the interview study, as well as five competences that were discussed at least as frequently as the survey-related competences.

*Personal qualities*, despite not having been identified as a top twenty-first century competence, was the most discussed competence in the interviews. Discussion incorporated sub-competences such as *curiosity*, *experimental approach*, *confidence*, *resilience*, *perseverance*, *reflection*, *attention to detail*, *playfulness with materials & products,* and *ability to think on one’s feet*. For example, Industrial Designer, ID1, spoke of the way *resilience* and *perseverance,* learned through Industrial Design education, had benefitted many areas of their life. They said,

[You learn] a way of sort of not becoming despondent or discouraged by little failures, which I think is really important because I think that’s one of the things that I learnt through Industrial Design that sort of, [pause] has run through the rest of my life.

And university lecturer, UT1, suggested that in order to encourage an *experimental approach*, students could be graded on how many genuine experiments they undertook in their projects, rather than on the production of high-quality presentation images or models.

The competences of *problem solving*, *STEM, creativity, interpersonal skills*, *A*rt, and *environmental sustainability knowledge* were also all discussed at length by the majority of interviewees. Additionally, their important sub-competences of *real-life problems*, *empathy*, *understanding of product function*, *manufacturing knowledge*, *materials knowledge*, *drawing*, and *beauty and aesthetics* were also all discussed at length. Comments included the following. Vocational education student, TS1, prioritised *environmental sustainability knowledge* over all else. They said, “I’ll mention the sustainability again because I think that’s what should be primarily focussed on”. Design teacher association representative, A1, spoke of Industrial Design education as a way of providing students with the agency and ability to tackle *real life problems*. They said, “We are hopefully empowering and supporting a load of young people who feel like they can come up with solutions or do something, just to make the world better”. While university student, US1, expressed a passion for *materials* and *manufacturing knowledge*. They stated, “a huge area which I’m really interested in, which is amazing, that’s super important, would be materials sciences and understanding different materials and their properties and how to use them in manufacturing”.

*Critical thinking*, *digital skills*, *entrepreneurial capability*, and *communication skills* were also discussed at substantial levels in the interviews. Particular attention was given to the sub-competence of *design software including 3D modelling software*. For example, vocational education student, TS1, described their acquisition of entrepreneurial skills when they said, “you learn how to operate a business, you learn how to contact people in industry, you learn how to make things, sell things”.

While Industrial Designer ID1 valued the communication skill of *listening*, stating, “you’ll do well if you’re able to listen very deeply and closely, and quickly synthesise information”. Over two thirds of interviewees discussed *entrepreneurial capability* and *communication skills* in detail and made an average of 3–4 mentions of *adaptability/flexibility*.

*Ethical understanding* was not included in the list of top twenty-first century competences used in this study, yet it was discussed more frequently by these interviewees than several of the top twenty-first century competences. For example, university leader, UL2, identified Industrial Design as a setting of strong tensions between actively ethical and actively unethical practice when they said, “I think Industrial Designers often struggle within their own minds about the ethics of Industrial Design, full-stop, because we kind of all know that we don’t need more consumer products”, but later reflected, “hopefully Industrial Design education produces graduates... as socially responsible problem solvers.... I think it is a trait of most graduates, I really do”.

Over half of the interviewees discussed each of the top twenty-first century competences of *collaboration* and *cultural literacy.* The final competence from the top twenty-first century competences list, *innovation capability,* was discussed moderately in the interviews, and at a lower rate than the three competences of *organisation skills, spatial imagination*, and *research/investigation*.

## Discussion

Several competences were identified at high levels in Industrial Design education. The *personal qualities* of *curiosity*, *experimental approach*, *confidence*, *resilience*, *perseverance*, *reflection*, and *attention to detail* were all present in these Industrial Design stakeholder interviews. They were also valued by the twenty-first century competences authors. The Industrial Design competences authors had not identified *personal qualities* at levels found in this study. This finding is, therefore, an original contribution to new knowledge. It suggests that Industrial Design education has a previously unpromoted potential for imparting valuable twenty-first century *personal qualities* to students.

Both the interviewees and the survey respondents saw *problem solving* as a key defining feature of Industrial Design education. This is consistent with the views of many Industrial Design competences authors (Erkarslan et al., 2011; O*Net, 2021; Tatlisu & Kaya, 2017; WDO, 2018; Yang, et al., 2005). *Problem solving* is also one of the most important twenty-first century competences (Adesida & Karuri-Sebina, 2013; Ananiadou & Claro, 2009; Donnelly & Wiltshire, 2014), so this finding suggests that *problem solving* is an important component of the value and twenty-first century relevance of Industrial Design education.

The combined analysis of the interviews and surveys suggested that *STEM* skills are vital to Industrial Design education but that the ideal Industrial Designer needs a broad but shallow knowledge of *STEM.* For example, interviewee ID1 spoke about designers having broad knowledge of many areas but being a “master of nothing”. They also said, “if you need to get something done you can draw on that little background you’ve got... little bit of understanding to talk the same language as the specialists”.

This idea is reminiscent of Brown and Wyatt’s (2010) concept of the “T-shaped person”.

Despite the fact that *Art* was extracted from *STEAM* for analysis and reporting, many interviewees mentioned *STEM* and *Art skills* in the same sentence, suggesting that Industrial Design education is a blend of *Art* and *STEM* (a *STEAM* discipline). For example, secondary teacher and design teacher association representative, A2, explained,

[Students] are thinking through more than the surface aesthetics of a product, they’re actually looking to how it functions. And that’s a very complex brain activity but it’s one that gives the mathematical student with a bent to creativity, a career path, and a really exciting one because of the breadth of Industrial Design.

Rhode Island School of Design (2018), and Taylor (2016) find that *STEM* is made more relevant when combined with Art to become *STEAM. This suggests* that Industrial Design education is a valuable vehicle for making the *STEM* education prioritised by governments, more relevant and engaging to students.

The vast majority of interviewees believed *creativity* was a crucial Industrial Design education competence. *Creativity* was similarly endorsed by the university survey cohort but less so by the secondary survey cohort. This was thought to be related to the “technical” and “making” priorities of the many secondary Design Technology teachers who responded to the survey. Creativity was the top twenty-first century competence identified in the literature (Education Council, 2019; EU, 2019; Frey & Osborne, 2017; Hajkowicz et al., 2016) and it was highly valued by the Industrial Design competences authors (Erkarslan, et al., 2011; Gunes, 2012; Lewis & Bonollo, 2002; O*Net, 2021; WDO, 2018; Yang et al., 2005). This suggests that the provision of creativity education is an important way that Industrial Design education provides value and relevance to twenty-first century students.

The top twenty-first century competence of *interpersonal skills* was not a surveyed competence. However, interviewees saw *interpersonal skills* as crucial in enabling Industrial Designers to connect with and manage relationships with peers, allied specialists, clients and end users. *Interpersonal skills* was also firmly prioritised by the twenty-first century competences authors (OECD, 2019; RAI & NBN, 2016; Voogt & Roblin, 2012). This suggests that Industrial Design education provides extensive twenty-first century value in this area. The sub-competence of *empathy* was of paramount importance to the interviewees. For example, vocational education student, TS2, explained the importance of Industrial Designers connecting with product end users. They said, “Like engineering, I reckon you probably get taught a bit, but they mainly make things work, whereas we connect more with the person who uses it, so we’re like the bridging gap between the user and the existing product”. This emphasis on *empathy* aligns with the views of many Industrial Design education theorists (Erkarslan et al., 2011; Lewis & Bonollo, 2002; NASAD, 2020; Tatlisu & Kaya, 2017; Yang et al., 2005) and twenty-first century authors (Bradlow, 2015; EU, 2019; Hajkowicz et al., 2016; Leadbeater, 2016).

*Environmental sustainability knowledge* was highly valued by participants. It was represented at broadly equivalent levels in the interviews, the survey study and the twenty-first century *life* literature. However, it was rarely mentioned in the twenty-first century competences literature and was mentioned moderately in the Industrial Design competences literature. This lower presence in the twenty-first century competences literature might reflect the input of corporations and governments in much of this literature (Voogt & Roblin, 2012; WEF, 2016a; 2018) and might result from these organisations prioritising economic measures of value over social and environmental wellbeing. Interviewees often spoke of environmental problem solving, thus linking the competence of *environmental sustainability knowledge* with the valuable twenty-first century competence of *problem solving*. Some interviewees also valued the competence of *environmental sustainability knowledge* for its potential to teach students about the environmental stories behind consumer products used in their daily lives. For example, interviewee A2 said,

[Environmental sustainability knowledge]’s not taught anywhere else and without that understanding . . . they won’t think about the pros and cons of what they’re consuming, and I think it’s important to have it as part of education considering its social, cultural, environmental, financial, legal, ethical impact on everybody’s life. I mean yes, we’re a visual society but we’re also a consumer society and Industrial Design plays a major role in that.

This is likely to be a key way that Industrial Design education provides value and twenty-first century relevance to students who do not go on to become Industrial Designers.

*Critical thinking* was of relatively high importance to the interviewees, survey respondents and twenty-first century competences authors. It was slightly less valued in the Industrial Design competences literature. However, it is possible that *critical thinking* was somewhat overrepresented in the interviews because of its prominence in various Australian school curricula (ACARA, 2021; Board of Studies NSW, 2013; VCAA, 2017a, VCAA, 2017b). Nevertheless, professional Industrial Designers were one of the groups most likely to discuss *critical thinking,* suggesting it belongs firmly in the Industrial Design curriculum.

The competence of *digital skills* was measured at roughly equivalent levels in the interviews, the surveys, the twenty-first century competences literature, and the Industrial Design competences literature. This suggests that Industrial Design education teaches *digital skills* at a relevant and appropriate level. However, the *digital skills* learning measured in the two studies did not entirely align with projections from the twenty-first century competences literature. The twenty-first century competences literature mentioned *virtual reality* (Gartner, 2017; Higgins, 2017)*, artificial intelligence* (Hajkowicz, 2016; WEF, 2020), the *Internet of Things* (Cerwall et al., 2018; Gartner, 2017; Lueth, 2018), the use of generative software (Autodesk, 2018), and programming capability (NBN & RIA, 2016; WEF, 2020) frequently but these topics were discussed very infrequently or not at all by the interviewees. This suggests they are areas that may need to be expanded in Australian Industrial Design education.

*Entrepreneurial capability* was valued more strongly by the interviewees than by the survey respondents. This might have been due to infrequent but regular programming of entrepreneurial learning at the university level and very infrequent but still regular programming of entrepreneurial learning at secondary level. *Entrepreneurial capability* was moderately less emphasised by the interviewees than by the twenty-first century competences authors. This suggests that Industrial Design learning would benefit from increased entrepreneurial training as promoted by Zhao (2012), Gunes (2012), and the European Union (2019). Because Industrial Design education is intimately entwined with commercialism, it is an ideal curriculum for imparting valuable entrepreneurial capabilities to twenty-first century students.

The top twenty-first century competence of *communication skills* was not included in the survey, but the interviewees discussed *communication skills* at broadly similar levels to the twenty-first century competences authors and the Industrial Design competences authors. Professional Industrial Designers, Industrial Design experts, and university staff and students were the most likely interviewees to discuss the importance of *communication skills*. This suggests that *communication skills* are crucial to the discipline of Industrial Design but that this fact is less well-known to non-experts. The types of *communication* skills discussed by interviewees included *cross-cultural communication skills*, *presentation skills*, *persuasion skills*, *negotiation skills*, *listening skills,* and *visual communication skills*. For example, Industrial Designer, ID2, explained the relevance of cross-cultural communication to many Australian Industrial Designers when they said, “we’re in Australia and a lot of suppliers are in China, so cultural differences and proximity challenges, all that sort of thing, so yeah, communication is an absolutely huge one”. Industrial Designer, ID1, echoed the views of Buchanan (1990), when they said, “I think it’s something that designers bring to a lot of projects is as the negotiator”.

The competence of *adaptability/flexibility* was measured at much higher levels in the surveys than the interviews. It was also much more valued by the twenty-first century competences authors than the Industrial Design competences authors. Explanations were not found for these disparities although it’s possible that the competence of *adaptability/flexibility* is present in Industrial Design education without it being something that readily comes to mind during discussions.

Although *ethical understanding* was not one of the very top twenty-first century competences, it was present in many classrooms and curricula and was prominent in many interviewee’s thoughts about Industrial Design education. For example, secondary teacher and Design association representative, A2, felt strongly about providing ethical Industrial Design education. They said, “[Students] need to be cognizant of how these things are made and what resources they’re using, and what personnel they’re using, in what country and culture they’re being made.” The high importance of *ethical understanding* in the interviews may have partly resulted from the imperative for education settings to deliver morality education (Education Council, 2019; EU, 2018), however *ethical understanding* is also recognised as an important component of Industrial Design education by the Industrial Design competences authors (Lewis & Bonollo, 2002; NASAD, 2020; Tatlisu & Kaya, 2017; Yang et al., 2005).

*Collaboration* was raised at approximately the same level in these interviews as in the survey study and the Industrial Design competences literature. However, the research participants endorsed *collaboration* far less than the twenty-first century competences authors did. Several interviewees mentioned the challenges of working collaboratively in education, saying things like, “The main problem at uni, is you always had the people that did all the work and the people that did none of the work.” (interviewee ID2), and, “this year I had a group project with someone who was very difficult, and you know, I’m difficult in ways, but I found that we really clashed” (interviewee US2). Combined analysis of the interviews and surveys indicated that *collaboration* must be taught in balance with independent work skills, so this partially accounts for the discrepancy. Interviewees classified as Industrial Design experts were most likely to discuss *collaboration,* and interviewees from education institutions were least likely to discuss it. This suggests the high importance of *collaboration* to the profession, as well as the difficulty of delivering collaboration skills within individualistic assessment systems.

*Cultural literacy* was observed at similarly moderate levels in the interviews, the surveys, the twenty-first century competences literature, and the Industrial Design competences literature. This suggests that it is taught at relevant levels in Industrial Design learning settings. Interviewees discussed *cultural literacy* in relation to understanding of one’s own and other cultures, as well as in relation to design history. Industrial Designer, ID2, spoke of the importance of, “trends, and themes, and... fashion or what people are drawn to and what’s coming up next, to imagine those things that don’t yet exist... and being aware of things, like outside your bubble, outside your existence”. However, university lecturer, UT1, expressed regret that design history seemed less prominent in Industrial Design education now than it once was. The non-surveyed competences of *organisation skills, spatial imagination,* and *research & investigation* were all spoken of at a similar level to *cultural literacy.*

*Innovation capability* was raised at much lower levels in the interviews than was seen in the survey responses, and at moderately lower levels than was seen in the twenty-first century competences literature. The reasons for this were unclear. However, results suggest that Industrial Design education is well equipped to impart the valuable twenty-first century competence of *innovation capability,* but that *innovation capability* is not the most important aspect of Industrial Design learning.

### Recommendations

Synthesis of the research findings elicited the following recommendations. Recommendations relating to ways that Industrial Design education could be used to impart valuable twenty-first century competences to students at a range of levels are described as follows. Industrial Design education should be recognised by policy makers and education leaders as a valuable way of delivering twenty-first century competences, and of educating students about the products that surround them; whether students go on to become Industrial Designers or not. Industrial Design education should be recognised for its ability to impart valuable *personal qualities* to students. It should also be recognised as one of the most important vehicles used for delivering crucial *entrepreneurial capabilities* to students at a range of levels. Industrial Design education is an ideal format for entrepreneurial learning given that it provides a structure for identifying problems, opportunities, and needs, and eventually producing valuable outcomes. Further to this, Industrial Design education should be one of the top means of delivering *STEAM* education. *STEAM* education is likely a more engaging model for delivering valuable *STEM* skills and knowledge (RISD, 2018; Taylor, 2016).

Recommendations relating to ways that Industrial Design education should be amended to better impart valuable twenty-first century competences to students are as follows. Firstly, Industrial Design assessment structures at all levels should be reviewed to ensure they reward acquisition of the most important competences. A good example of this approach is university lecturer, UT1’s, suggestion that in order to encourage an experimental approach, students be graded on how many genuine experiments they undertake, rather than on the production of high-quality presentation images and models. Secondly, collaboration skills should be overtly taught within Industrial Design education. Frameworks that support fairness, process, and interpersonal relationships in relation to *collaboration* should be provided to students to support the acquisition of this challenging but important competence. Finally, although there is anecdotal evidence of many tertiary Industrial Design courses already incorporating programming, IoT, AR, VR, and emerging technologies, the results of this study suggest that some tertiary courses may not be including these areas very often. Therefore, it is recommended that tertiary, and possibly secondary, courses are reviewed to assess for the presence of sufficient programming, IoT, AR, VR, and emerging technologies content.

One general recommendation relating to *environmental sustainability knowledge* was also derived from this research. We recommend that *environmental sustainability knowledge* be recognised as an important twenty-first century competence in the literature. The twenty-first century *life* authors refer to it repeatedly (Davidson, 2005; Meadows et al., 1972; Randers, 2012; Turner, 2008) and the majority of participants in this research considered it as a crucial knowledge area. Environmental protection will be crucial across the globe over coming decades (Davidson, 2005; Meadows et al., 1972; Randers, 2012, Turner, 2008) so all twenty-first century students should study it extensively in order to be able to problem solve in this area as they progress into the workforce. *Environmental sustainability knowledge* needs to be listed alongside *creativity*, *problem solving*, *critical thinking,* and all the other top twenty-first century competences.

### Potential future research

The rating tool developed for this study could be adapted for a range of related purposes. For example, Artefact Analysis or Document Analysis could be conducted on student Industrial Design outcomes or assessment materials. Alternatively, the survey structure could be adapted for use by educators and education leaders to assess the twenty-first century relevance of different Design or non-Design curricula in their institutions. It is noted that the synthesis of knowledge coming from multidisciplinary areas continues to be a challenge, however if design education is to continue to be relevant to current public and political debates, it must actively readjust its focus to give students opportunities to learn more about both their discipline and themselves (Bernardi & Kowaltowski, 2010). This is relevant for all design disciplines and links to Ergenoglu (2015) highlighting key competences for universal design education; namely, building empathy, social and legal awareness, awareness regarding the physical environment, universal design knowledge, inclusive and universal design settings, best practices, typological studies and developing new approaches and design solutions (Ergenoglu, 2015); all of which could be studied in further detail mapped against other design disciplines besides Industrial Design. Additionally, as a substantial amount of design research explores the views and status of design educators, design students, and designers (Dorst, 2015; Findeli, 2001; Kimbell, 2012; Nichols, 2013; van Diggelen & Bruns Alonso, 2015), further research is recommended in the area of non-designer views on what design is and what it should be. This would be very instructive in relation to what design audiences want from designers.

## Conclusion

Combined analysis of the interviews and surveys portrayed Industrial Design as a discipline that uses intellectual, interpersonal, and physical capabilities; and that can deliver value and twenty-first century relevance through a broad competence base including *creativity, problem solving, critical thinking, communication, digital skills, STEM skills*, and *innovation capability*.

Industrial Design education in Australia appears to deliver some twenty-first century competences at particularly high levels and these are, *interpersonal skills, ethical understanding*, and *organisation skills,* as known by the Industrial Design competences authors; as well as *environmental sustainability knowledge; adaptability/flexibility*; and *personal qualities* such as *curiosity, resilience, confidence, perseverance*, and *reflection*; a fact not widely documented in the existing Industrial Design competences literature. Industrial Design education is therefore likely to be an important vehicle for delivering these increasingly important soft skills and educating future students about increasingly urgent environmental sustainability principles.

This research has measured specific items of value and relevance in Industrial Design education, and it has covered a range of Industrial Design education levels not previously investigated as a whole. This research characterised Industrial Design education as imparting a very broad skills and knowledge base and a large number of crucial twenty-first century competences. Industrial Design education is likely to deliver particularly strong capabilities in the twenty-first century competences of *personal qualities* (including sub-competences such as *curiosity, resilience, confidence, perseverance,* and *reflection*); *interpersonal skills* (especially *empathy*); *environmental sustainability knowledge*; *organisation skills*; and *ethical understanding*. Industrial Design education may benefit from more overt teaching of *collaboration* skills, the nurturing of further *entrepreneurial skills,* and further inclusion of IoT, VR, AR, software development, and other emerging technologies. *Environmental sustainability knowledge* appears to be becoming increasingly important to Industrial Design students; and Industrial Design education appears to impart many important general life skills. It is recommended that Industrial Design education be used to deliver important twenty-first century competences from primary school level through to university level, and that its value be recognised through grants, public funding, and policy reform.

## Appendix

See Tables 5, 6.

**Table 5:** Most in-demand twenty-first century competences according to the literature

Most in demand twenty-first century competences	Level of importance as indicated by frequency of mentions in the literature
Creativity	Extremely high importance 40 mentions
Problem solving	Very high importance 34 mentions
Collaboration	High importance All a similar level of importance ~ 26–29 mentions
Innovation capability	
Digital skills/connectivity	
Entrepreneurial capability	
Critical thinking	
Adaptability/flexibility	
Communication skills	
STEM or STEAM	
Interpersonal skills	
Cultural literacy	Moderate to high importance 16(+ 7?) mentions
Global outlook	Significantly important 13–16 mentions
Leadership	
Literacy	
Learning to learn	
Resilience/perseverance	
Numeracy	Moderately important OR perhaps very important for some people but not all people 8–11 mentions
Technological knowledge	
Judgement /decision making	
Citizenship	
Research skills	
Risk-taking	
Initiative/agency	
Emotional intelligence	
Persuasion/negotiation	
Financial Literacy	
Self-esteem/confidence	
Ethical understanding	
Human–machine collaboration	Somewhat important OR perhaps important for some people but not all people 4–7 mentions
Empathy	
Software development/programming	
Organisation skills/project management	
Curiosity	
Environmental sustainability knowledge	
Complexity/uncertainty management	
Experimental approach	
Presentation skills	
Media literacy	
Technical knowledge	

Adesida and Karuri-Sebina (2013), Ananiadou and Claro (2009), Anderson et al. (2017), Australian Government (2018), Australian Government (2018b), Autor (2015, p12,27), Beitz (2015), Bradley (2015), Bradlow (2015), Brynjolfsson and McAfee (2012), Callander (2015), Chui et al. (2015), Commonwealth of Australia (2015), de Voogt et al. (2011), den Hollander (2015), Donnelly and Wiltshire (2014), Durrant-Whyte (2015), Education Council (2015), Education Council (2019), EU (2019), Frey and Osborne (2017), FYA (2017a), FYA (2017b), FYA (2017c), Gratton (2015), Green et al. (2015), Hajkowicz et al. (2016), Halal et al. (2016), Hampson et al. (2017), Herson (2012), Hinds et al. (2004), Innovation and Science Australia (2017), Kuratko (2009), Lambert (2017), Leadbeater (2016), NEA (1999), OECD (2019), P21 (2007), Perez (2001), RAI (2016), RISD (2018), Scott (2015), Tee and Xu (2015), United Nations (2013), UNESCO (2015), United States House of Representatives (2013), Van Laar et al. (2017), Voogt and Roblin (2012), Watson (2016), WEF (2016a), WEF (2016b), Wright et al. (2013), Zhao (2012), Zhou (2017)

**Table 6:** Top industrial design competences with references

Top Industrial Design competences	Level of importance as indicated by frequency of mentions in the literature
STEM	Erkarslan et al. (2011), Goatman and Moody (2014), Lewis and Bonollo (2002), NASAD (2020), O*Net (2021), Tatlisu and Kaya (2017), WDO (2018), Yang et al. (2005)
Entrepreneurial capability	Erkarslan et al. (2011), Goatman and Moody (2014), Gunes (2012), Lewis and Bonollo (2002), NASAD (2020), O*Net (2021), WDO (2018), Yang et al. (2005)
Problem solving	Erkarslan et al. (2011), Lewis and Bonollo (2002), NASAD (2020), O*Net (2021), WDO (2018), Tatlisu and Kaya (2017), Yang et al. (2005)
Creativity	Erkarslan et al. (2011), Gunes (2012), Lewis and Bonollo (2002), O*Net (2021), Tatlisu and Kaya (2017), WDO (2018), Yang et al. (2005)
Interpersonal skills	Erkarslan et al. (2011), Lewis and Bonollo (2002), NASAD (2020), O*Net (2021), WDO (2018), Yang et al. (2005)
Empathy	Erkarslan et al. (2011), NASAD (2020), Nichols (2013), O*Net (2021), Tatlisu and Kaya (2017), Yang et al. (2005)
Art skills	Erkarslan et al. (2011), Goatman and Moody (2014), NASAD (2020), O*Net (2021), Yang et al. (2005)
Communication skills	Lewis and Bonollo (2002), NASAD (2020), O*Net (2021), Tatlisu and Kaya (2017), Yang et al. (2005)
Digital skills / connectivity	Erkarslan et al. (2011), NASAD (2020), O*Net (2021), Tatlisu and Kaya (2017), Yang et al. (2005)
Design process knowledge	Erkarslan et al. (2011), NASAD (2020), Lewis and Bonollo (2002), Tatlisu and Kaya (2017), Yang et al. (2005)
Understanding of product function	Erkarslan et al. (2011), Goatman v Moody (2014), O*Net (2021), NASAD (2020), Tatlisu and Kaya (2017)
Beauty & aesthetics	Erkarslan et al. (2011), O*Net (2021), NASAD (2020), Tatlisu and Kaya (2017), Yang et al. (2005)
Manufacturing knowledge	Goatman and Moody (2014), Lewis and Bonollo (2002), O*Net (2021), NASAD (2020), Yang et al. (2005)
Industrial Design is an intellectual pursuit	Goatman and Moody (2014), Lewis and Bonollo (2002), O*Net (2021), NASAD (2020), Yang et al. (2005)
Literacy	Lewis and Bonollo (2002), O*Net (2021), NASAD (2020), Tatlisu and Kaya (2017), Yang et al. (2005)
Critical thinking	Lewis and Bonollo (2002), O*Net (2021), NASAD (2020), Yang et al. (2005)
Environmental sustainability knowledge	NASAD (2020), O*Net (2021), WDO (2018), Yang et al. (2005)
Innovation capability	Erkarslan et al. (2011), Gunes (2012), WDO (2018), Yang et al. (2005)
Collaboration	Erkarslan et al. (2011), O*Net (2021), NASAD (2020), Yang et al. (2005)
Ethical understanding	Lewis and Bonollo (2002), NASAD (2020), Tatlisu and Kaya (2017), Yang et al. (2005)
Cultural literacy	O*Net (2021), NASAD (2020), Tatlisu and Kaya (2017), Yang et al. (2005)
Organisational skills / project management	Lewis and Bonollo (2002), O*Net (2021), Tatlisu and Kaya (2017), Yang et al. (2005)
Research skills	O*Net (2021), NASAD (2020), Tatlisu and Kaya (2017), Yang et al. (2005)
Drawing	NASAD (2020), O*Net (2021), Yang et al. (2005)
Learning to learn	NASAD (2020), Tatlisu and Kaya (2017), Yang et al. (2005)
Personal qualities	Lewis and Bonollo (2002), O*Net (2021), Yang et al. (2005)
Design software including CAD	Erkarslan et al. (2011), O*Net (2021), Yang et al. (2005)
Materials knowledge	O*Net (2021), Tatlisu and Kaya (2017), Yang et al. (2005)
Working independently	Lewis and Bonollo (2002), O*Net (2021), Yang et al. (2005)
Adaptability / flexibility	NASAD (2020), O*Net (2021), Yang et al. (2005)
